# Rapid detection of *Echinococcus* species by a high-resolution melting (HRM) approach

**DOI:** 10.1186/1756-3305-6-327

**Published:** 2013-11-14

**Authors:** Guilherme Brzoskowski Santos, Sergio Martín Espínola, Henrique Bunselmeyer Ferreira, Rogerio Margis, Arnaldo Zaha

**Affiliations:** 1 Programa de Pós-Graduação em Biologia Celular e Molecular, Universidade Federal do Rio Grande do Sul, Caixa Postal 15005, CEP 91501-970, Porto Alegre, RS, Brazil; 2 Programa de Pós-Graduação em Genética e Biologia Molecular, Universidade Federal do Rio Grande do Sul, Caixa Postal 15053, CEP 91501-970, Porto Alegre, RS, Brazil; 3 Centro de Biotecnologia, Universidade Federal do Rio Grande do Sul, CEP 91501-970, Porto Alegre, RS, Brazil

**Keywords:** *Echinococcus* species, High-resolution melting (HRM), Genotyping

## Abstract

**Background:**

High-resolution melting (HRM) provides a low-cost, fast and sensitive scanning method that allows the detection of DNA sequence variations in a single step, which makes it appropriate for application in parasite identification and genotyping. The aim of this work was to implement an HRM-PCR assay targeting part of the mitochondrial cox1 gene to achieve an accurate and fast method for *Echinococcus* spp. differentiation.

**Findings:**

For melting analysis, a total of 107 samples from seven species were used in this study. The species analyzed included *Echinococcus granulosus* (n = 41) and *Echinococcus ortleppi* (n = 50) from bovine, *Echinococcus vogeli* (n = 2) from paca, *Echinococcus oligarthra* (n = 3) from agouti, *Echinococcus multilocularis* (n = 6) from monkey and *Echinococcus canadensis* (n = 2) and *Taenia hydatigena* (n = 3) from pig. DNA extraction was performed, and a 444-bp fragment of the cox1 gene was amplified. Two approaches were used, one based on HRM analysis, and a second using SYBR Green Tm-based. In the HRM analysis, a specific profile for each species was observed. Although some species exhibited almost the same melting temperature (Tm) value, the HRM profiles could be clearly discriminated. The SYBR Green Tm-based analysis showed differences between *E. granulosus* and *E. ortleppi* and between *E. vogeli* and *E. oligarthra.*

**Conclusions:**

In this work, we report the implementation of HRM analysis to differentiate species of the genus *Echinococcus* using part of the mitochondrial gene cox1*.* This method may be also potentially applied to identify other species belonging to the Taeniidae family.

## Findings

The cyclophyllidean family Taeniidae is generally accepted to be composed of two valid genera, *Taenia* Linnaeus, 1758 and *Echinococcus* Rudolphi, 1801. Currently, there are nine recognized species within the genus *Echinococcus*, and four of them have medical significance: *Echinococcus multilocularis, Echinococcus granulosus*, *Echinococcus oligarthra* and *Echinococcus vogeli*[1]. The taxonomic position of *E. granulosus* has been recently revised, and species status was attributed to some of its 10 genotypes (G1-G10): *E. granulosus sensu stricto* (G1-G3), *E. equinus* (G4), *E. ortleppi* (G5), and *E. canadensis* (G6-G10) [2]. Another two species are now included in the *Echinococcus* genus, *E. shiquicus* and *E. felidis*[3,4]. Additionally, many of these species coexist in the same area, as is the case for *E. granulosus* and *E. ortleppi* and for *E. oligarthra* and *E. vogeli*[5,6]. The genus *Taenia* consists of approximately 50 species that are difficult to identify. Most of these are of medical and veterinary importance and lead to systemic (cysticercosis and coenurosis) and intestinal infections (taeniasis) [7,8].

As a genotyping tool, the high-resolution melting (HRM) method has been used for the rapid differentiation of influenza A subtypes, for identification of the *Cryptococcus neoformans*-*Cryptococcus gatti* complex, and for parasites of the phylum Platyhelminthes and Protozoa [9-13]. For *Trypanosoma cruzi,* the HRM method has been implemented based on the amplification of a 383-bp DNA fragment of the cytochrome b gene (cyt b), allowing the effective differentiation of 14 genotypes and showing that the use of amplicons derived from mitochondrial genes is reliable and sufficiently robust for use in HRM [14].

The aim of this work was to develop an HRM-PCR assay targeting part of the mitochondrial cox1 gene to achieve a rapid, accurate, and low cost diagnostic method for detecting species belonging to the *Echinococcus* genus. In this work, we report the successful implementation of HRM analysis to differentiate species of the genus *Echinococcus* using part of the mitochondrial cox1 gene.

A total of seven species belonging to the Taeniidae family were used in this study (Table 1). The DNA extraction of *E. granulosus* and *E. ortleppi* was performed from protoscoleces, using proteinase K, and from germinal layer [5,15]. DNA samples from the remaining species were obtained using the PureLinkTM Genomic DNA Kit (Invitrogen, USA), following the manufacturer’s instructions.

**Table 1:** Species examined, the samples location, host and the number of isolates

			**SYBR Green Tm-based**			**HRM analysis**			**Validation test**		
**Species**	**Location**	**Host**	**Number of isolates**	**Tm**	**SD**	**Number of isolates**	**Tm**	**SD**	**Number of isolates**	**Tm**	**SD**
*E. ortleppi*	Southern Brazil	Bovine	18	82.70	0.08	6	83.35	0.02	32	81.70	0.03
*E. granulosus*	Southern Brazil	Bovine	15	84.04	0.13	4	83.72	0.07	26	82.30	0.05
*E. oligarthrus*	Northern Brazil	Agouti	3	82.56	0.05	3	82.70	0.07	-	-	-
*E. vogeli*	Northern Brazil	Paca	2	83.30	0.05	2	84.10	0.14	-	-	-
*E. multilocularis*	Europe	Monkey	-	-	-	6	82.70	0.07	-	-	-
*E. canadensis* (G7)	Southern Brazil	Pig	-	-	-	2	82.05	0.03	-	-	-
*T. hydatigena*	Southern Brazil	Pig	-	-	-	3	81.95	0.07	-	-	-

The melting temperatures (Tm) and standard deviations (SD) obtained in the SYBR Green Tm-based, HRM analyses and validation test are shown.

A 444-bp fragment of the cytochrome c oxidase subunit I (cox1) gene [16], was amplified using the primers 5′-TTTTTTGGGCATCCTGAGGTTTAT-3′ (forward) and 5′-TAAAGAAAG AACATAATGAAAATG-3′ (reverse) in the following reaction mixture: 50 ng of DNA template, 5 mM dNTP, 5 pmol of each primer, 1.2 mM MgCl_2_, 1 U Taq polymerase (Invitrogen, USA), 20 mM Tris–HCl (pH 8.4), 50 mM KCl, in a total volume of 20 μL. The PCR reactions were performed in a 7500 thermal cycler (Applied Biosystems) under primer annealing touchdown strategy [17]. All PCR reactions were carried out in technical triplicates with non-template controls (NTCs).

In an attempt to discriminate and accurately identify differences in all seven species, we used two approaches. SYBR Green Tm-based analysis*,* in which the dissociation step was obtained from the 7500 Applied Biosystems machine (with SDS software) using 1X SYBR green dye (Invitrogen, USA) and increasing the temperature from 60°C to 95°C at ramping increments of 0.2°C/s (Table 1). A second approach employed the HRM technique (Table 1), that was carried out with SYTO 13 (Life technologies), a DNA dye that does not preferentially bind to GC- or AT-rich sequences, does not increase the melting temperature, and has a minimal inhibitory effect on PCR [18,19], at a 2.5 μM final concentration, in a final volume of 10 μL, using the Eco Real-Time PCR System (Illumina ®). In this assay, melting was conducted by increasing the temperature from 60°C to 99°C at ramping increments from 0.1°C/s. The HRM analysis was carried out using the Eco software (version 3.0) with normalization regions between 79.1–79.4°C and 87.1–87.4°C. A difference melting curve was produced with *E. ortleppi* as baseline.

To validate HRM-cox 1 profiles, 58 isolates of *Echinococcus* spp. collected from hydatid cysts of bovine livers and lungs were analyzed. DNA from protoscoleces and germinal layer were used to evaluate the usefulness of this technique to differentiate *Echinococcus* species. The HRM was carried out using Rotor-gene Q 2plex System (Roche ®) as described above, in attempt to show that this method is reproducible with alternative systems. As reference curves of HRM, a known sample for *E. granulosus* and *E. ortleppi* was used.

One positive amplicon of each sample derived from PCR-HRM that displayed distinct curve shapes were sequenced. Sequence quality assessment and assembly was performed using DNAStar software (version 8.1.3). After PCR amplification, confirmation of the species was performed by a homology search against reference sequences using the Basic Local Alignment Search Tool (BLAST) program hosted by the National Centre for Biotechnology Information (http://www.ncbi.nlm.nih.gov).

**Figure 1:**
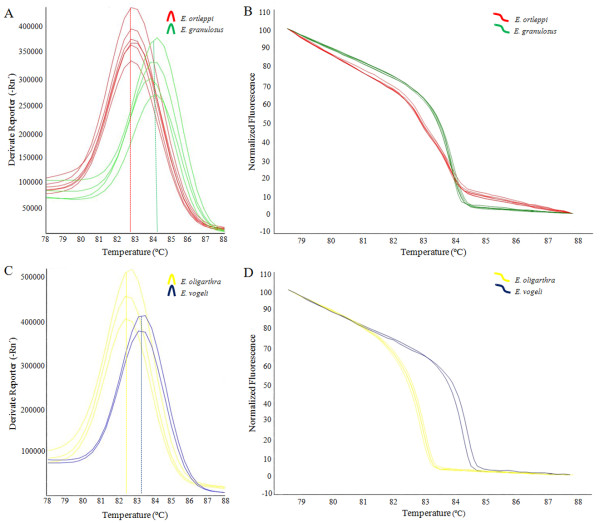
**Comparative SYBR Green Tm-based and HRM curve analyses. (A)** SYBR Green Tm-based and **(B)** HRM analyses of *E. ortleppi* (n = 6) and *E. granulosus* (n = 4). **(C)** SYBR Green Tm-based and **(D)** HRM analyses of *E. vogeli* (n = 2) and *E. oligarthra* (n = 3). The dotted lines show the Tm values from the analyzed species.

A DNA fragment of 444 bp from the cox1 mitochondrial gene was amplified in all analyzed samples and no amplification was observed in the control reactions (NTCs). With SYBR Green Tm-based and HRM analyses, very low Tm variability was observed (Table 1). *E. granulosus* and *E. ortleppi* melting curves showed a difference in the Tm of 1.34°C (Table 1; Figure 1A). Likewise, *E. vogeli* and *E. oligarthra* showed a difference of 0.74°C (Table 1; Figure 1C). The differences in the Tm were at least nine and eight times higher than the respective standard deviation (SD), respectively (Table 1). These results refer to the total number of samples used, although in Figure 1, for comparison, only the curves of the isolates that were also used in the HRM approach are presented. Similarly, the Tm obtained from the HRM analysis, showed a difference of 0.37°C between *E. granulosus* and *E. ortleppi* and a difference of 1.40°C between *E. vogeli* and *E. oligarthra*. In this analysis, the SDs were five and ten times lower than the differences in Tm, respectively (Table 1; Figure 1B and D).

**Figure 2:**
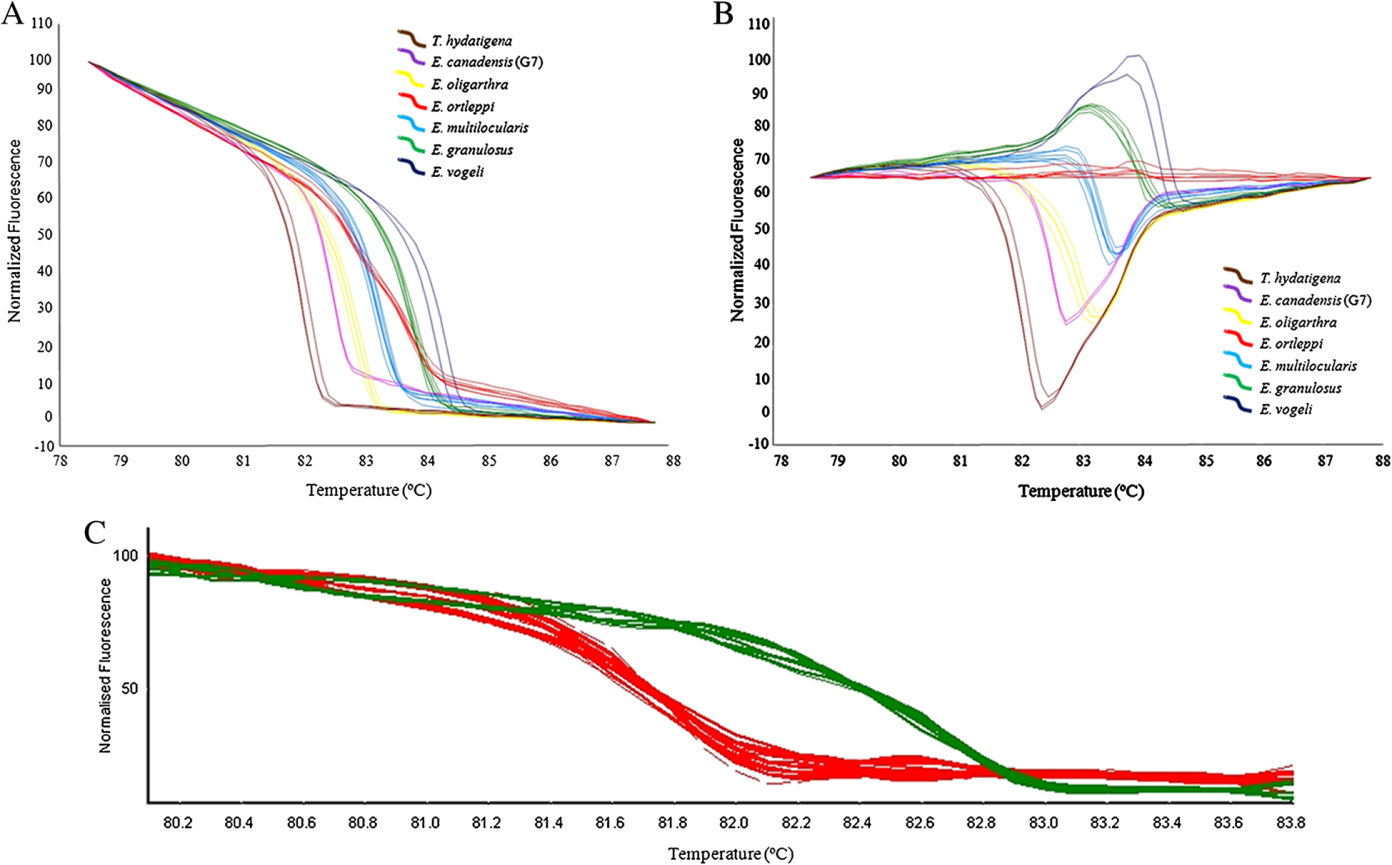
**High-resolution melting (HRM) from the seven species belonging to the Taeniidae family, and validation test. (A)** Normalization curves derived from the raw data plots. **(B)** Difference curves derived from the normalization data using *E. ortleppi* as baseline. **(C)** HRM profile obtained from *E. ortleppi* and *E. granulosus* samples in the validation test.

In the HRM analysis, a specific profile for each species analyzed was observed in both the standard and the difference curve (Figures 2A and B, respectively). Despite the close Tm value observed for some species, the HRM profiles provide a clear and undoubted discrimination among them. See, for example, the comparisons between *E. multilocularis* and *E. ortleppi* or between *E. canadensis* (G7) and *E. oligarthra* (Table 1; Figure 2A and B). In our validation test, from the HRM profiles of the cox1 gene, thirty-two samples were correctly identified as *E. ortleppi* and twenty-six of the samples obtained were identified as *E. granulosus sensu stricto* (G1) (Table 1; Figure 2C). The results obtained by PCR-HRM for all the tested samples were confirmed by sequencing the amplicons. BLASTn analysis showed identity values greater than 99% for all samples.

We show, for the first time, that it is possible to distinguish *E. granulosus* from *E. ortleppi* as well as *E. vogeli* from *E. oligarthra* using the Tm obtained from the melting analysis of cox1. Furthermore, we compared this species with *E. multilocularis*, *E. canadensis* and *T. hydatigena*, and discriminated them by HRM, that is a useful and advantageous method that can be routinely employed in endemic regions of echinococcosis. This observation will be useful for additional HRM analyses of the Taeniidae family and, thus, will serve as a basis for subsequent interpretations.

The development of fast and effective tools for *Echinococcus* species identification has been the subject of numerous studies. For instance, morphological differences and the cut-off value for adult and larval hook were determined to distinguish *E. granulosus sensu lato* isolates [20,21]. However, the larval rostellar hook morphometry method is dependent on the presence of protoscoleces. In contrast, HRM analyses allow the use of DNA extracted from any parasite material as we showed in the validation test (Figure 2C).

In an attempt to implement an *Echinococcus* genotyping tool, a multiplex PCR (mPCR) test was used, using eleven pairs of primers, and it successfully differentiated the species of the *Echinococcus granulosus* complex [22]. Here, we present a technique that only requires a single pair of primers that amplify the cox1 gene, providing a quick, closed-tube and gel-free detection method for species belonging to the *Echinococcus* genus (Figure 2A and B). In our procedure, the identification of the species of the genus *Echinococcus* took approximately 6 hours. Recently, the application of HRM analysis was reported for genotyping *E. granulosus sensu lato* in Iran to distinguish G6 from the G1 and G3 genotypes, but it did not show good results in distinguishing G1 from G3 [23].

In conclusion, we propose the use of HRM of the cox1 gene as a routine method to distinguish *Echinococcus* species in epidemiological surveys or in basic research (Figure 2). This method may also be applied to identify other species belonging to the Taeniidae family. Finally, we believe that the HRM method could be implemented using a smaller amplicon product when the goal is to identify minor differences, such as in the case of closely related genotypes.

## Abbreviations

HRM: High-resolution melting; NTCs: Non-template controls; BLAST: Basic local alignment search tool; Tm: Melting temperature; SD: Standard deviation.

## Competing interests

There are no conflicts of interest.

## Authors’ contributions

GBS participated in the design of the study, and in the HRM experiments. Wrote the manuscript. SME participated in the SYBR Green Tm-based experiments, helped to draft the manuscript. HBF participated in the design of the study and helped to draft the manuscript. RM participated in HRM experiments and helped to draft the manuscript. AZ participated in the design of the study and helped to draft the manuscript. All authors read and approved the final version of the manuscript.
